# The molecular biology and therapeutic potential of Nrf2 in leukemia

**DOI:** 10.1186/s12935-022-02660-5

**Published:** 2022-07-29

**Authors:** Atefeh Khodakarami, Sara Adibfar, Vahid Karpisheh, Shiva Abolhasani, Pooya Jalali, Hamed Mohammadi, Jamshid Gholizadeh Navashenaq, Mohammad Hojjat-Farsangi, Farhad Jadidi-Niaragh

**Affiliations:** 1 Immunology Research Center, Tabriz University of Medical Sciences, Tabriz, Iran; 2 Student Research Committee, Tabriz University of Medical Sciences, Tabriz, Iran; 3 Department of Immunology, School of Medicine, Kermanshah University of Medical Sciences, Kermanshah, Iran; 4 Non-Communicable Diseases Research Center, Alborz University of Medical Sciences, Karaj, Iran; 5 Noncommunicable Diseases Research Center, Bam University of Medical Sciences, Bam, Iran; 6 Bioclinicum, Department of Oncology-Pathology, Karolinska Institute, Stockholm, Sweden; 7 Department of Immunology, School of Medicine, Bushehr University of Medical Sciences, Bushehr, Iran; 8 Department of Immunology, Faculty of Medicine, Tabriz University of Medical Sciences, Tabriz, Iran; 9 Research Center for Integrative Medicine in Aging, Aging Research Institute, Tabriz University of Medical Sciences, Tabriz, Iran

**Keywords:** Leukemia, Transcription factor, Nrf2, Chemotherapy

## Abstract

NF-E2-related factor 2 (Nrf2) transcription factor has contradictory roles in cancer, which can act as a tumor suppressor or a proto-oncogene in different cell conditions (depending on the cell type and the conditions of the cell environment). Nrf2 pathway regulates several cellular processes, including signaling, energy metabolism, autophagy, inflammation, redox homeostasis, and antioxidant regulation. As a result, it plays a crucial role in cell survival. Conversely, Nrf2 protects cancerous cells from apoptosis and increases proliferation, angiogenesis, and metastasis. It promotes resistance to chemotherapy and radiotherapy in various solid tumors and hematological malignancies, so we want to elucidate the role of Nrf2 in cancer and the positive point of its targeting. Also, in the past few years, many studies have shown that Nrf2 protects cancer cells, especially leukemic cells, from the effects of chemotherapeutic drugs. The present paper summarizes these studies to scrutinize whether targeting Nrf2 combined with chemotherapy would be a therapeutic approach for leukemia treatment. Also, we discussed how Nrf2 and NF-κB work together to control the cellular redox pathway. The role of these two factors in inflammation (antagonistic) and leukemia (synergistic) is also summarized.

## Introduction

Leukemia is a group of blood and bone marrow cancers that can be fatal at any age [1]. Scientists classify leukemia into different groups based on its speed of progression and the type of involved cells. Leukemia is classified into acute and chronic groups according to how fast it progresses. Acute leukemia is more common in children than chronic leukemia, usually in adults [2]. Another type of leukemia classification is the kind of affected white blood cells; lymphocytic and myelogenous. Concerning the mentioned classifications, scientists categorized leukemia into four significant groups acute lymphocytic leukemia (ALL), chronic lymphocytic leukemia (CLL), acute myeloid leukemia (AML), and chronic myeloid leukemia (CML) [3, 4]. The symptoms of leukemia are usually unclear and non-specific and include lymphadenopathy, thrombocytopenia, fever, fatigue, weight loss, bone pain, bruising, and bleeding [5, 6]. Leukemia's most common treatment options are chemotherapy, radiation therapy, immunotherapy with interferon, and hematopoietic stem cell transplantation [7, 8]. However, these standard therapies can cause various complications like tumor lysis syndrome and serious infections (due to immunosuppression and recurrence of the disease) [9]. Some patients do not respond to current treatments and become resistant to them. Accordingly, identifying new therapeutic targets to improve survival rates for patients with leukemia is very important.

The human NF-E2-related factor (Nrf2) protein is a master regulator of ARE-bearing genes that regulate and induce the expression of cytoprotective and antioxidant genes to maintain redox homeostasis. More than 600 genes are regulated by the Nrf2 signaling pathway, with more than 200 encoding cytoprotective proteins linked to inflammation, cancer, neurological disorders, aging, cardiovascular disease, and other serious illnesses. Furthermore, mounting evidence is that the Nrf2 signaling pathway is dysregulated in many malignancies, resulting in abnormal Nrf2 expression. Also, due to its ability to regulate the expression of many genes that modulate apoptosis, cell survival, proliferation, inflammation, tumor metastasis, and angiogenesis, the transcription factor Nrf2 is critical for cancer cell survival [10, 11]. In normal cells, following exposure of cells to oxidative and electrophilic stresses, Nrf2 is released into the cytoplasm and transported to the nucleus. Subsequently, nuclear Nrf2 transcribes several genes involved in the oxidative response, which protects against DNA damage and inflammation [12, 13].

On the other hand, aberrant expression and function of Nrf2 defend cancer cells against radio- or chemotherapy. Nrf2 overactivation also causes oncogenic cell proliferation and survival in many cancers, especially leukemia [14–16]. This review summarized the current knowledge about Nrf2 and its essential role in leukemia progression and drug resistance. In addition, we also described recent targeting mechanisms and novel therapeutic approaches in the final part of the study. Therefore, future studies could reveal how targeting this factor may increase the sensitivity of leukemic cells to chemotherapy.

## NRF2

### Nrf2 structure

**Fig. 1:**
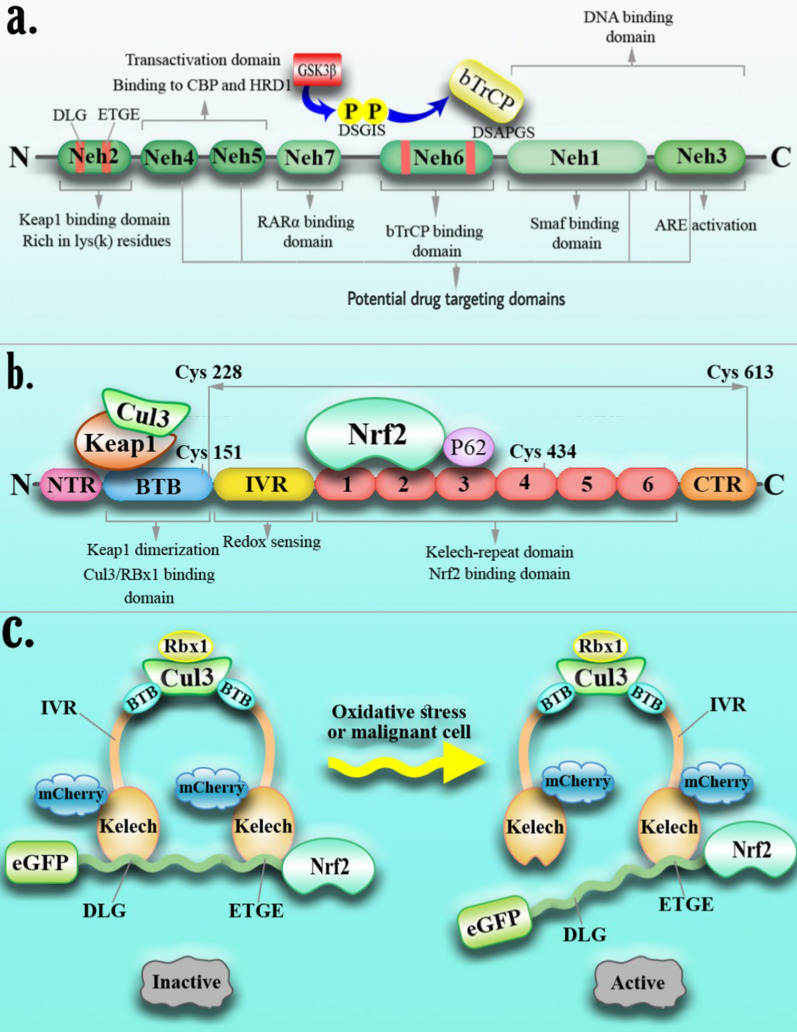
Interaction of Keap1 and Nrf2. Keap1 (an Nrf2 inhibitor) has three major domains: BTB, IVR, and Kelch. The BTB domain is involved in Keap1 dimerization, CUL3 binding, and ROS sensing by C151 (Cys). The Kelch domain consists of six Kelch repeats, which bind to Nrf2 and P65 proteins. The IVR domain is also a redox-sensing domain (**a**). Nrf2 has seven domains; Neh2 is the Keap1 binding domain through DLG and ETGE motif interaction. Neh4, 5, 3 domains are critical for transactivation. Moreover, Nrf2, through the Neh3 domain, binds to the ARE region of the chromosome. The Neh6 domain has serine (S) residues in the DSGIS motif. GSK3β phosphorylates this motif and supports recognition by βTrCP, and Neh1 interacts with small MAF proteins (**b**). Under normal conditions, Nrf2 has been suppressed through binding to the Kelch domain of Keap1, but in the presence of ROS or malignant cells, the release of Nrf2 from Keap1 occurs, and the transfer of Nrf2 to the nucleus alters the pattern of gene expression (**c**)

Human Nrf2 (also known as a nuclear factor, erythroid 2-like 2; NFE2L2) gene is located at locus 2q31.2 on chromosome 2 and encodes multiple isoforms produced by alternative splicing. Nrf2 is a member of the cap′n′collar family of basic leucine zipper (bZip) transcription factors and consists of various functional domains, including seven highly conserved Nrf2-ECH homology domains (Neh1-Neh7), among which Neh1, 3,4, and 5 are associated with Nrf2 activation, and Neh2, 6, and 7 are involved in the blockade of Nrf2 protein [17–19]. Neh1 is a basic leucine-zipper (bZip) containing domain that attaches to the musculoaponeurotic fibrosarcoma (Maf) protein and promoter region of DNA. Neh3 is located at the C-terminus of the protein and is involved in Maf recognition elements (MARE) activation [20, 21]. Neh2 and Neh6 have critical roles in Nrf2 ubiquitination and proteasomal degradation. The Neh2 domain in the N-terminal site binds Keap1 (Kelch-like ECH-associated protein 1) and Nrf2 adaptor protein in a specific E3 ubiquitin ligase complex. Neh2 domain possesses two key motifs, DLG and ETGE, with high and low affinity for Keap1 [22, 23] (Fig. 1a). Keap1 consists of three domains: BTB, IVR, and KLECH. The BTB domain at the N-terminal is dimerized with Cul3 and forms a hinge and lock-like inhibitory structure following Nrf2 inhibition. The IVR domain is an oxidation condition sensor at the Keap1 C terminal adjacent to the KLECH [24, 25] (Fig. 1b). The researchers used CRISPR/Cas9 to deactivate the Nrf2 gene in A549 cells by cutting the Neh4 and Neh5 domains in lung cancer cells which altered the Nrf2 nuclear export signal (NES) region. The protein is phenotypically prevented from traveling into the nucleus after translation. A549 cells with this gene deletion were shown to have a decreased proliferation phenotype in tissue culture and to be more sensitive to chemotherapeutic drugs such as cisplatin and carboplatin. These findings were supported by xenograft mouse models in which homozygous mutant cells proliferate slower than wild-type cells, even in the absence of pharmacological therapy. The growth of tumors was stopped for 16 days, and the size of tumors in samples treated with CRISPR-directed gene editing and chemotherapy decreased significantly [26]. Accordingly, we can suppress the Nrf2 activator domains in cancer therapy by using the CRISPR/Cas9 technique, which impedes Nrf2 attachment. Also, in gene targeting, one ought to pay attention to the characteristics of Nrf2 within the sort of cancer to what effect this inhibition has on the manner of development or worsening cancer. Moreover, in leukemia target therapy, intervention in any of the pathways involved in signaling and Nrf2 activation seems to be a promising strategy.

### Nrf2 signaling 

**Fig. 2:**
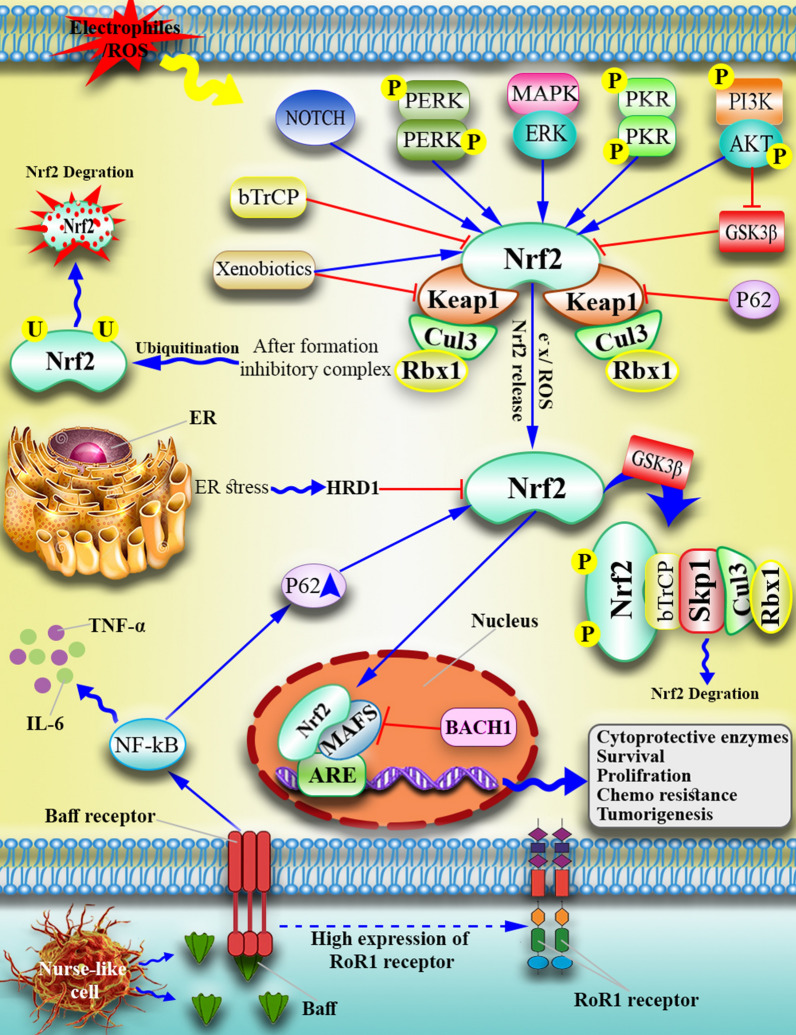
Nrf2 signaling pathway in the malignant cells. In normal conditions, three E3 ubiquitin ligases suppress Nrf2 by forming an inhibitory complex (Keap1-CUL3-RBX1, β-TrCP-SKP1-CUL1-RBX1, and HRD1). However, in leukemic cells or under oxidative stress, Nrf2 is released from this negative regulator and activates the transcription of target genes after forming heterodimers with sMAF. Moreover, Nurse-like cells produce Baff in the malignant cell microenvironment that induces ROR1 and Baff receptor expression on the malignant cells, leading to an inflammatory cytokine (NF-κB, IL6, TNF) increase inside the leukemic cells and recruitment of P65p, which binds to Keap1, and releases Nrf2 from the Keap1-CUL3-RBX1 inhibitory complex

Under normal cellular conditions, Nrf2 is repressed in the cytoplasm by inhibitory molecules such as Keap1, which leads to ubiquitination and proteasomal degradation through cullin3 (CUL3), which contains E3 ubiquitin ligase. However, oxidative stress conditions lead to structural changes in Keap1, which affect disulfide bonds and cause Nrf2 to be released from Keap1 [27, 28] (Fig. 1c). The released Nrf2 is translocated to the nucleus and forms a heterodimer with small MAF proteins. As a transcription factor, the Nrf2/MAF heterodimer binds to the ARE region of the chromosomal enhancer and activates the transcription of hundreds of genes, including detoxifying and cytoprotective genes. Transcribed genes reduce ROS expression and maintain cell homeostasis [29–31] (Fig. 2). Under normal conditions, Nrf2 maintains cellular redox homeostasis and regulates cell proliferation, which is the molecular basis of Nrf2's role in cancer prevention [13]. But, there are several pathways to explain how the Nrf2 signaling pathway is constitutively active in certain cancers: (a) Oncogenic *Myc*, *K-Ras*, and *B-Raf* mutations promote Nrf2 transcription via mitogen-activated protein kinases (MAPKs) [32] (b) Somatic mutations in Keap1, Nrf2, or Cul3 that disrupt the Nrf2/Keap1 interaction [33] (c) Exons in Nrf2 mRNA are lost, resulting in Nrf2 mutants that do not interact with Keap1 [34] (d) Keap1 epigenetic DNA methylation lowers Keap1 expression [35] (e) PTEN tumor suppressor gene mutations and epigenetic changes raise Nrf2 levels [36] (f) Proteins that compete with Keap1 include BRCA2 partner and localizer (PALB2), dipeptidyl peptidase 3 (DPP3), Wilms tumor gene on the X chromosome (WTX), p21, and p62 [37] (g) Keap1 cysteine succination due to loss-of-function mutation [38].

Multiple signaling pathways are implicated in both Nrf2 suppression and activation, allowing for fine-tuning Nrf2 signaling under different physiological situations.

#### Nrf2 activation

Nrf2 turnover is high in terms of homeostasis and lack of oxidative response [39]. However, in oxidative reactions like ROS-activating signaling, the existence of electrophiles, and autophagy disorder, electrons bind to sulfhydryl groups at the Keap1 surface, which changes the structure of the Keap1 protein. Following the Keap1 structure alteration, Nrf2 is dissociated from Keap1 and activated [40]. Nrf2 stabilization occurs as a result of different mechanisms; in the first model, the "hinge and lock" model (a) (Keap1 separation), peripheral electrons attach to the cysteine roots (Cys273, Cys288) of Keap1 protein in electrophilic and oxidative stress conditions, which leads to structural changes of Keap1 and results in modification-dissociation of Nrf2 protein, which increases Nrf2 accumulation in the nucleus (shown with anti-Nrf2 immunoblot assay). So, the reduction of Cys273 and Cys288 roots of Keap1 (in the IVR domain) induces ubiquitin-proteasomal degradation of Nrf2. These alterations prevent the formation of the "hinge and latch" model, disrupt the proteasome degradation of Nrf2, and stabilize Nrf2 in the cytoplasm. Researchers highlighted that repression of Nrf2 activity needs both Keap1 roots (Cys273 and Cys288). These roots play a crucial role in the regulatory pathway [41, 42]. Furthermore, in a luciferase reporter test, a Keap1 mutant lacking the IVR domain lost its ability to decrease Nrf2 activity. In contrast, an Nrf2 model protein (Neh2-green fluorescent protein [GFP]) can be sequestered in the cytoplasm by the mutant Keap1. As a result, an alternate model emerged in which oxidative alteration or mutation of the cysteine residues in Keap1 impacts the rapid turnover mechanism of Nrf2 [42]. The second, "Cul3 separation" model (b), explains Nrf2 turnover in response to cellular redox state alterations in a Cul3-dependent mode. A coordinated cascade including an E1 ubiquitin-activating enzyme, an E2-conjugating enzyme, and an E3 ubiquitin ligase leads to ubiquitination. Cullin-RING E3 ligases have a RING motif that arranges E2 binding, allowing ubiquitin to transfer from the E2 to the target protein's lysine (Nrf2), leading to target protein degradation. Cysteine151 (Cys151) of Keap1 as an adaptor of Cul3 binding to Nrf2. Peripheral electrons affect this bond and disrupt the interaction between Keap1 and Cul3 proteins. Dissociation of Keap1 and Cul3 hinders Nrf2 ubiquitination, and finally, Nrf2 stabilizes freely in the cytoplasm [43].

#### Autophagy-dysregulation and Nrf2

P62 protein (or sequestosome 1 [SQSTM1]) is an essential regulator of Nrf2, which acts in a Keap1-dependent manner through a non-canonical pathway of NF-κB [44, 45]. Generally, P62, as a scaffold protein, binds to the cargo and directs it to the autophagosome for degradation [46, 47]. The P62 competes with Nrf2 for binding to the Keap1. As p62 contains a high-affinity motif for Keap1, it sequesters Keap1, brings it to the autophagosome, and consequently rescues Nrf2 from Keap1-dependent degradation [44, 48, 49]. Notably, the P62-mediated Nrf2 regulation axis disrupts autophagy and increases NF-κB expression levels [44, 45] (Fig. 2). Recent studies have proven the synergistic effect of elevated Nrf2 and NF-κB, which ultimately disrupt the autophagy pathway and involve inflammatory cell responses [50].

#### Suppression of Nrf2

##### Keap1-dependent regulation of Nrf2

Kelch, a Drosophila actin-binding protein, is the closest homolog to Keap1, suggesting that Keap1 is an Nrf2 cytoplasmic effector. The cytoplasmic protein Keap1 was unknown to the researchers. At first, they thought Nrf2 prevents its own activation in an unknown mechanism. Also, they thought that the DNA-binding activity of Nrf2 is dramatically increased in response to electrophilic chemicals. Their findings show that Keap1 suppresses Nrf2 and that changes in Keap1 cysteine roots in the presence of electrons boost Nrf2 activation. To investigate the molecular mechanisms that activate Nrf2 and hence transduce oxidative stress signals, Itoh et al. first examined the structure and functional domains of the Nrf2 protein, a comparison of the human, chicken, and mouse Nrf2 molecules revealed that they were cross-species homologs. Consequently, they are referred to hereafter as Nrf2. As a result of this comparison, they discovered an experimental localization of a domain inside the cNrf2 molecule capable of negatively controlling its activity in transfected cells. They discovered that this domain is linked to a new cytoplasmic protein that inhibits Nrf2's transactivation potential. Researchers gave this new protein the name Keap1 [51]. Also, data showed that Neh2 appears to be a structurally composite domain divided into two subregions upon closer analysis. The 32-amino-acid amino-terminal region of Neh2 is rich in hydrophobic residues and shares many similarities with the amino-terminal regions of Nrf1 and the C. elegans Skn-1 transcription factors. On the other hand, the carboxy-terminal half of Neh2, which corresponds to amino acid residues 33–73, is hydrophilic and not conserved among CNC family members. Because the carboxy-terminal half of Neh2 is conserved across species, it could represent a key functional domain unique to Nrf2 [51]. Keap1 diametrically binds to the Neh2 domain (ETGE and DLG motifs) and negatively affects Nrf2 activation. Also, it is an essential adaptor protein for the cullin3 (Cul3) containing E3 ubiquitin ligase [40, 51, 52]. Cul3 assembles with Keap1 and makes a framework that provides the basis for binding E3 ligase (RBX1) to the Keap1/Nrf2 dimer, whereby seven lysine residues of Nrf2 are ubiquitinated [53]. In the following, AAA + ATPase p97 extracts ubiquitylated Nrf2 from the complex and then transports it to 26 s proteasome for the degradation process [54–56] (Fig. 2). Keap1 deficiency causes constitutive activation of Nrf2-dependent genes, although postnatal mortality in Keap1-deficient mice is reversed in mice lacking Nrf2 and Keap1 [56, 57]. Furthermore, the Nrf2- Keap1 connection is hampered by cyclin-dependent kinase inhibitor 1 (p21), a prominent target of the tumor suppressor p53. P21 interacts directly with the DLG and ETGE domains of Nrf2, preventing Keap1 binding in response to oxidative stress [58]. Post-translational modifications of Nrf2, such as phosphorylation and glycation, can influence Keap1-mediated regulation of Nrf2 activity. In response to oxidative stress, protein kinase C (PKC) phosphorylates Nrf2 at Ser40, preventing Keap1 binding and promoting Nrf2 nuclear accumulation [59]. AMPK, the cellular energy sensor, phosphorylates Nrf2 at Ser550, a residue within the canonical nuclear export signal, promoting its nuclear accumulation [60]. Glycation of Nrf2 promotes Keap1-mediated Nrf2 degradation and reduces Nrf2 interaction with sMAF proteins. In addition, Fructosamine-3-kinase (FN3K) phosphorylates the associated sugars to reverse Nrf2 glycation [61].

Generally, we found that under constant oxidative stress and abundant electrons, structural changes arise in Keap1 that induce the Nrf2 release from the Keap1-CUL3-RBX1 inhibitory complex, which shows the inhibition of the "hinge and lock" model formation. Moreover, post-translational modifications can influence Keap1-mediated regulation of Nrf2 activity.

##### b-TrCP-dependent regulation of Nrf2

Glycogen synthase kinase-3 (GSK-3) phosphorylates and inactivates the glycogen synthase enzyme. Two different isoforms were identified for GSK-3: alpha (GSK-3α) and beta (GSK-3β). GSK3β is involved in cellular metabolism, nerve cell growth, body pattern formation, and other cell signaling pathways [62, 63]. Keap1-independent activation of GSK3β leads to Nrf2 inhibition [64]. Neh6 consists of two motifs, including DSGIS and DSAPGS, which are recognized separately by the F box domain of the β-transducing repeat-containing protein (βTrCP) [65, 66]. GSK3β induces phosphorylation of the DSGIS motif at Ser344 and Ser347 of Neh6 and enhances the affinity of βTrCP for its target, Nrf2 [66–68]. In the nucleus, β-TrCP binds to the ubiquitin ligase complex SKP1-CUL1-RBX1 E3 via its F-box motif and ubiquitylates nuclear Nrf2 independent of Keap1, which results in Nrf2 inhibition [65, 66] (Fig. **2**). GSK-3 activity is suppressed in response to insulin and growth factors by the phosphatidylinositol-3 kinase (PI3K) and protein kinase B (PKB/AKT) axis. Thus, PI3K/AKT increases Nrf2 activity while GSK-3 suppresses it. Surprisingly, the phosphatase and tension homolog (PTEN) works as a redox sensor and a significant antagonist of the PI3K/AKT signaling cascade [69].

In addition to Keap1, these studies introduce Nrf2 inhibitors such as GSK-3β and βTrCP, which act downstream of GSK-3β. In this case, the use of a therapeutic formulation to increase the affinity of these enzymes and make them more effective in the possible suppression of Nrf2 can be helpful.

##### Hrd1-dependent regulation of Nrf2

Ubiquitin-Protein Ligase E3 Synoviolin (also known as HMG-CoA reductase degradation protein 1 (Hrd1)) is a crucial ubiquitin ligase in the endoplasmic reticulum (ER) that is known as a regulator of Nrf2 in cirrhosis. Unfolded protein response (UPR) is induced by increased ROS levels and ER stress in the cirrhotic liver. Accumulated unfolded proteins in the ER lumen bind to Bip molecules, resulting in Bip detachment from the IRE1a monomer, and then free IRE1a starts an endogenous pathway [70, 71]. IRE1a cuts unspliced XBP1u mRNA and produces XBP1s. Subsequently, XBP1s transcriptionally activate the expression of genes involved in ER-related degradation (ERAD), such as HRD1. HRD1 interaction with the Nrf2 Neh4 and Neh5 domains mediates Nrf2 degradation in ER stress conditions [72] (Fig. **2**). The control of Nrf2 by Hrd1 is less understood, as only limited studies of each system have been reported.

Recent studies highlight the role of Nrf2 in controlling cirrhosis, so targeting Nrf2 in cirrhosis may be promising. In addition, these studies also introduce another Nrf2 negative regulator (HRD1) that is activated during enfold protein accumulation or cirrhosis.

### Nrf2 function

Nrf2 activates the transcription of several genes, such as redox signaling, then protects cells against the effects of exogenous and endogenous insults such as xenobiotics and oxidative stress [73, 74]. Besides, several studies on Nrf2 confirmed it as an essential tumor suppressor, and its inhibition in mice increased cancer risk [75–78]. Researchers found that in Nrf2^−/−^ mice, detoxification of N-nitrosobutyl (4hydroxybutyl) amine (BBN) carcinogen was impaired and increased the risk of bladder and liver carcinoma. On the other hand, wild-type mice responded more strongly to oltipraz (inducing an enzyme phase 2 response) than Nrf2^−/−^ mice [75]. This study shows the opposite role of Nrf2 compared to previous studies and highlights that activation of this pathway signaling causes a reduction in the risk of liver and lung cancer. Also, following diesel exhaust exposure, Nrf2-null mice had an enhanced production of DNA adducts in the lung [79]. In Nrf2-null mice, acetaminophen-induced liver damage is more severe [80]. Smoking-induced emphysema is more common in Nrf2-null mice [81]. In Nrf2-null mice, bleomycin-induced lung fibrosis is exacerbated [82]. According to a recent study, Nrf2 protects against pulmonary fibrosis via controlling intracellular ROS levels and the balance of Th1 and Th2 cells [83]. In line with the previous studies, suppressing Nrf2 by siRNA in HepG2 cells (hepatocellular carcinoma cell line) increased colony formation, cell growth, migration, metastasis, and plasticity [76]. The above studies have shown that suppressing Nrf2 in leukemia can be promising and can also progress bladder and liver carcinomas.

**Fig. 3:**
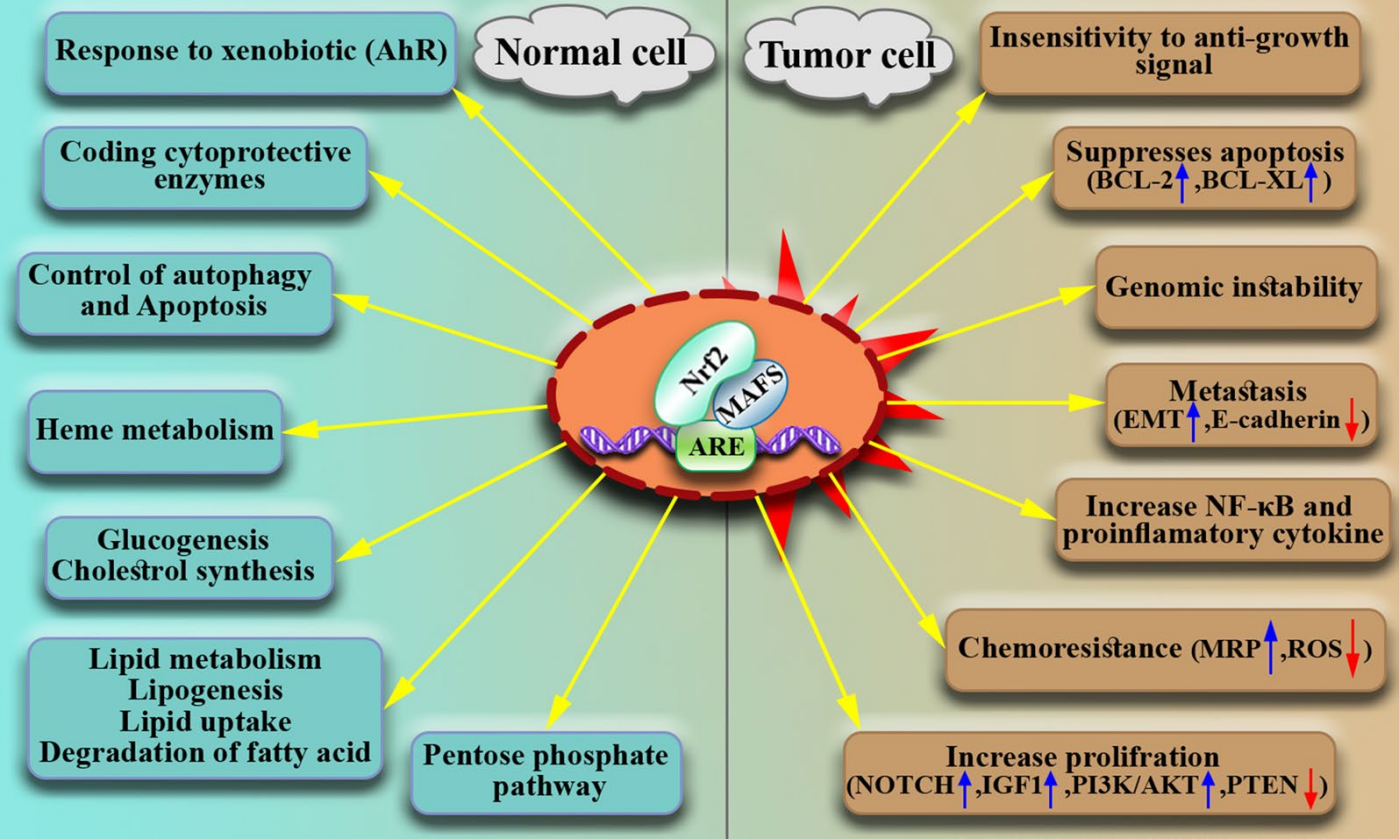
Dual function of Nrf2. Depending on cellular conditions, Nrf2 can play both an oncogenic role (in malignant cells) and a tumor suppressor role (in normal cells)

According to cancer type and stage, Nrf2 has different functions. Nrf2 acts as a double-edged sword as the Nrf2 signaling pathway can protect the growth and survival of both normal and malignant cells. Accordingly, Nrf2 can be considered a proto-oncogene, although more research is needed [84–86]. Generally, Nrf2 promotes cancer hallmarks, including angiogenesis, metastasis, proliferation, invasion, growth, survival, and resistance to chemotherapy and radiotherapy (Fig. 3).

#### Nrf2 as a gene inducer

Nrf2 quickly reduces the vulnerability of cells to oxidants and electrophiles by triggering the transcriptional activation of over a hundred cytoprotective genes, through suppression of ROS signaling, including the antioxidants such as ferritin, glutathione-S-reductase (GSR), glutamyl cysteine ligase-modulator (GCLM), and -catalytic (GCLC), phase-I drug oxidation enzyme NAD(P)H: quinone oxidoreductase 1 (NQO1), and cytoprotective enzyme heme oxygenase-1 (HO-1) [11, 14, 15, 87–89] (Fig. 2). Also, other transcription factors such as NF-κB and AP-1 are involved in Nrf2 expression regarding HO-1. In solid tumors like prostate cancer [90, 91], HO-1 is overexpressed at higher levels and correlated with increased tumor angiogenesis [88, 92–94].

#### Proliferation

Cell proliferation genes like NOTCH1, NPNT, BMPR1A, IGF1, ITGB2, PDGFC, VEGFC, and JAG1, have been shown to regulate Nrf2 [95, 96]. Some proteins, like PTEN phosphatase, are involved in the PI3K-AKT (PKB) signaling pathway, regulating cell survival. Previous studies have confirmed that in various diseases, including cancer and diabetes, the PI3K-AKT (PKB) signaling pathway is increased. Mutations in the PTENs and PI3K-AKT (PKB) pathways cause Nrf2 overactivation, which increases cell proliferation and cancer risk [97]. In addition, studies have shown the upregulation of Nrf2 and its target genes (AKR1c1, GCLC) in pancreatic cancer cell lines (Suit-2, MiaPaca-2, FAMPAC cells)[98]. This study showed more vital staining of cytoplasmic Nrf2 in most tumors than benign ducts. Silencing Nrf2 with a siRNA cut the cell lines' lifespan and growth rate, making them more sensitive to chemotherapy and radiotherapy. Nrf2 helps pancreatic cancer cell lines grow and become immortal [98], and targeting it in pancreatic adenocarcinoma is as promising as leukemia. Moreover, Nrf2 induces the differentiation of various cell types, including osteoclasts, adipocytes, hematopoietic stem cells (HSCs), and hepatocytes. Also, it is involved in the proliferation of airway basal stem cells (ABSCs), satellite cells (SCs), neuronal progenitor cells (NPCs), hepatocytes, and cancer cells [99]. In general, most studies confirm the role of Nrf2 in increased cell proliferation and introduce this process as a factor in treatment resistance.

#### Apoptosis

Nrf2 directly suppresses apoptosis by inducing the expression of BCL-2 and BCL-Xl [100]. Nrf2 also inhibits the intracellular apoptosis pathway by blocking cytochrome C release from mitochondria [101]. Studies on Nrf2 and its relation to apoptosis demonstrate that after treatment with cytotoxic agents such as cyclophosphamide or etoposide, Nrf2 represses intracellular and extracellular apoptosis pathways by suppressing caspase-7–3 activation [102, 103]. Therefore, upregulation of BCL-2 increases Nrf2 activity [104]. Nrf2 depletion using siRNA in periodontal ligament stem cells (PSCs) enhances caspase 3 and 9 and increases Bax at both protein and mRNA levels [105].

Conversely, activating Nrf2 with Sulforaphane in THP1 cells reduces mycobacterial infection and induces apoptosis in cell lines by activating caspase 3/7 independent apoptosis pathways [106]. In another study, researchers used Baicalin to induce apoptosis in H9c2 cells. Baicalin induced the Nrf2/HO-1 and HIF1a/BNIP3 pathways and increased apoptosis in H9c2 cells [107]. Although there are conflicting studies on the role of Nrf2 in inducing apoptosis, presented studies have shown that Nrf2 activity in cancer cells suppresses apoptosis.

#### Angiogenesis

Endothelial cells (EC) that line blood vessels have long been known to affect vascular health, repair, and illness (e.g., plaque formation in atherosclerosis). Endothelial cells have recently been discovered to function in tumor formation. Researchers discovered that pro-inflammatory signaling and spontaneous metastasis of lung cancers in mice could be stimulated by dysfunctionally activated endothelial cells (DEC). Furthermore, data showed that in DEC, Snail and Twist transcript levels increased, many pro-inflammatory NF-κB target genes (e.g., GMCSF, IL-8, IL-6, and E-selectin) were significantly enhanced, STAT3 phosphorylation increased, and the expression of leukocyte adhesion molecules VCAM1 and ICAM1 were increased simultaneously, which triggered endothelial-to-mesenchymal transition (EndMT) and metastasis. Conversely, healthy ECs stimulate vascular repair and prevent tumor invasiveness and metastasis [108]. Nrf2 plays a crucial role in forming mouse cerebral microvascular endothelial cells (bEnd.3). Under hypoxia conditions, Nrf2 expression increases temporarily in bEnd.3 cells induce the PI3K/Akt pathway while stimulating angiogenesis by increasing VEGF. Nrf2 blockage suppresses proliferation, endothelial tube formation, metastasis, and VEGF expression in hypoxia-induced bEnd.3 cells [109]. Thus, Nrf2 targeting brain strokes and leukemia can improve the healing process. Additionally, Nrf2 has a central role in the angiogenesis of glioblastoma. Blockage of Nrf2 signaling in glioblastoma decreases the expression of HIF-1a and VEGF due to angiogenesis [110, 111]. Moreover, another study showed that Nrf2 depletion in colon cancer xenograft-mouse models inhibited angiogenesis and suppressed VEGF and HIF-1a expression levels [112]. It has also been demonstrated that Nrf2 blockage significantly reduces the endothelial cells' survival, proliferation, and angiogenesis capacity in a posterior limb ischemia model[113]. Generally, most studies have proven that Nrf2 knockdown can reduce angiogenesis in xenograft-bearing mouse models. Blockage of Nrf2 can inhibit tumor growth by reducing the expression level of some genes involved in cell growth like HIF-1a, VEGF, PDGF, angiopoietin, and angiogenin [110, 112, 114].

Therefore, the role of Nrf2 in inducing angiogenesis in leukemia is consistent with other studies, and its targeting inhibits the development of new blood vessels.

#### Metastasis

Nrf2 plays a dual role in cancer cells. Nrf2 enhances EMT by regulating N-cadherin, E-cadherin, vimentin, Slug, and L1CAM [115, 116]. Nrf2 has a pro-invasive/metastatic role in the Colo357 cell line (pancreatic ductal adenocarcinoma) and HPDE cells (human pancreatic duct epithelial); it inhibits E-cadherin as well as reduces TGF-β1, induces Smad 2/3, and increases the JNK pathway in Colo357 and HPDE cells [115]. In addition, Nrf2 is involved in the metastasis of Eca-109 cells (squamous cell carcinoma) under hypoxic conditions [116]. It has also been reported that in non-small cell lung cancer (NSCLC), resistant to radiotherapy, the expression of Nrf2 and NOTCH1 increases synergistically. The inhibition of these two factors in A549 and H460 cell lines caused reduced E-cadherin and increased N-cadherin and matrix metallopeptidase (MMP)-2/9. Thus, targeting Nrf2 and NOTCH1 is a pivotal regulator of EMT that reduces radiotherapy resistance and metastasis in lung cancer [117, 118]. Moreover, Nrf2 released by Keap1 deactivation induced lung cancer metastases through suppression of Bach1 (another pivotal regulator of EMT) degradation in a Ho-1-dependent manner, so targeting Ho-1 or Nrf2 blockade can decrease lung cancer metastases [119]. In contrast, in in vivo research, DIM (3,3′-diindolylmethane) reduced DNMT expression and reversed Nrf2 CpG methylation status, resulting in increased expression of Nrf2 and Nrf2-target gene NQO1. TRAMP mice fed a DIM-supplemented diet had significantly reduced tumorigenesis and metastatic rates than the untreated control group. In prostate tissues, DIM increased apoptosis, decreased cell proliferation, and elevated Nrf2 and Nrf2-target gene NQO1 expression [120]. Another in vitro study on breast cancer cells showed that Nrf2 depletion in the MCF7 and MDA-MB-231 cell lines decreased proliferation and metastases by suppressing the RhoA/ROCK pathway [121–124]. Notably, decreased adhesion molecules and increased EMT are associated with metastasis in cancerous cells [125, 126].

In summary, Nrf2 is involved in metastasis, treatment resistance, and the progression of many cancers, including pancreatic cancer, squamous cell carcinoma, lung cancer, breast cancer, and leukemia.

#### The immunologic function of Nrf2

Nrf2 expression under normal conditions causes homeostasis of the immune system and prevents tumorigenesis, while Nrf2 supports cancer progression in cancerous cells [40]. Activating Nrf2 by inflammatory mediators such as prostaglandin, nitric oxide, and nitro fatty acids reduces inflammatory cytokines such as TNF α, IL-6, IL-1β, and NF-κB expression. Therefore, Nrf2 and NF-κB control the ROS level by reducing and increasing inflammation, respectively [127–132]. Accordingly, it has been demonstrated that downregulation of Nrf2 in mice is associated with persistent inflammation [127, 133, 134].

Nrf2 is involved in the functional development of CD8 + T cells through Glutathione (GSH) production [135, 136]. Since the naïve T CD8^+^ cells cannot produce GSH, macrophages produce GSH to help T cells develop their function. Accordingly, it has been shown that when Nrf2 has not expressed in bone marrow-derived macrophages, the functional evolution of CD8^+^ T cells is impaired due to low GSH expression [135]. *Nrf2-null* mice demonstrated higher levels of ROS and increased MDSC numbers than the wild type due to an abortive antigen recognition pathway and cellular energy metabolism in T CD8^+^ cells [78, 137–139].

#### Nrf2 and inflammation

**Fig. 4:**
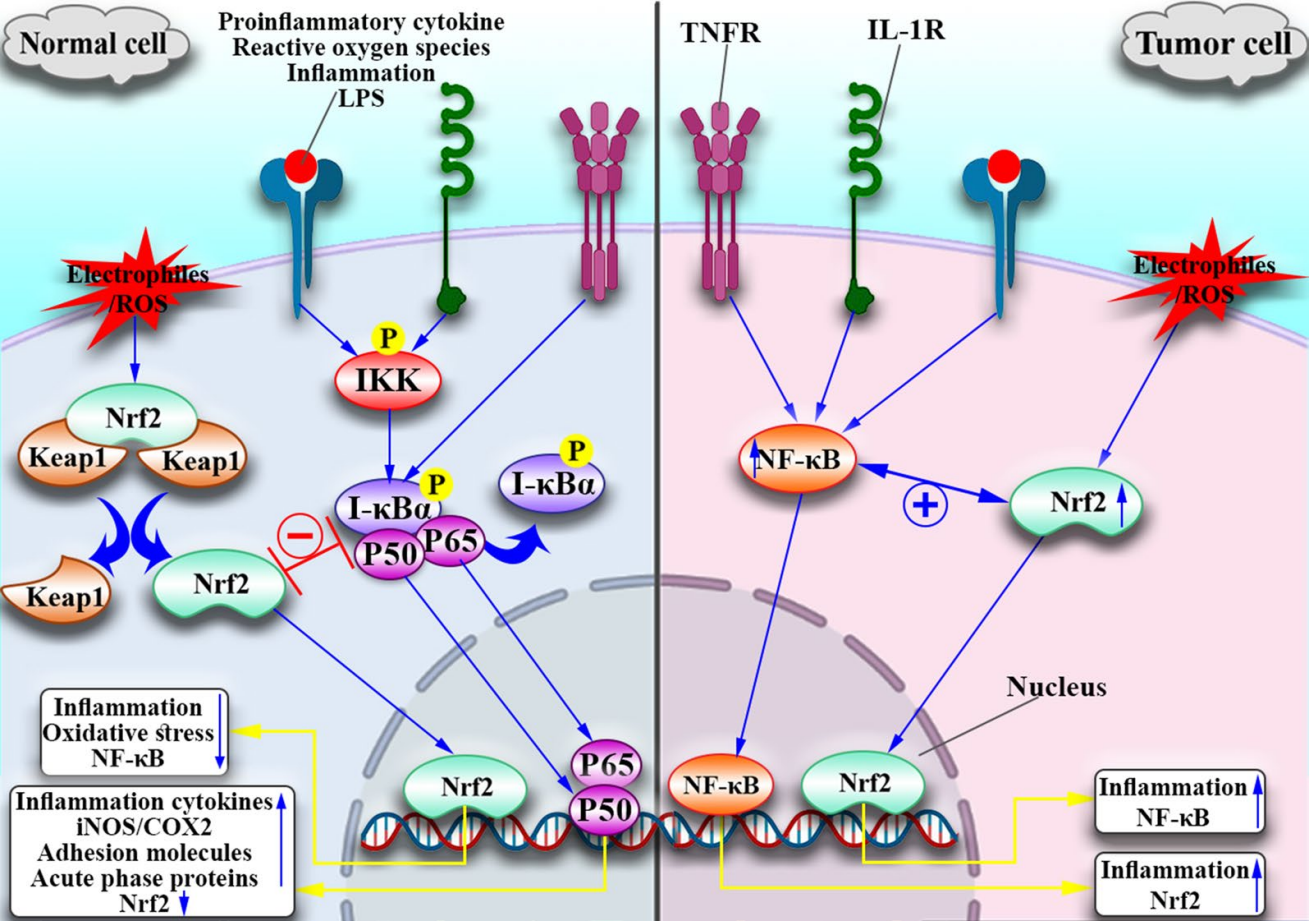
Cross-Talk Between Nrf2 And NF-κΒ. In response to NF-κΒ activators such as pro-inflammatory cytokines (IL-1, TNF-α), LPS, inflammation, and ROS, normal cells induce phosphorylation of IKKβ, which causes phosphorylation and degradation of IκBα (a negative regulator of NF-κΒ). Then NF-κΒ (P65/P50) migrates to the nucleus and triggers gene transcription involved in inflammation. However, Nrf2 suppresses NF-κΒ activation and inflammation directly through Keap1 attachment, or Ho-1 induces P65 suppression. Conversely, in malignant cells, Nrf2 and NF-κΒ act synergistically

To clarify the role of Nrf2 in inflammation, we should first discuss the cross-talk between Nrf2 and NF-κB (Fig. 4). In normal cells, the absence of Nrf2 increases NF-κB, which then enhances the production of cytokines, and inflammatory mediators (IL-1, IL-6, IL-10, TNF-α, COX, NO, iNOS), and adhesion molecules (ICAM-1, VCAM-1) [140, 141]. Nrf2 inhibits NF-κB expression in several ways. For example, in the presence of NF-κB, Keap1 released from the Nrf2 inhibitory complex then binds to IKKβ (IκBα inhibitor; IκBα is a negative regulator of NF-kB), causing ubiquitination and degradation of IKKβ (Fig. 4). Moreover, HO1 plays a role in heme metabolism by catalyzing the cleavage of heme's porphyrin ring into Fe2 + , CO, and biliverdin, which is then changed to bilirubin. These enzyme products directly suppress p65 (one of the NF-κB subunits) and reduce cell adhesion molecules [142]. Furthermore, analyses of *Nrf2-null* MEFs demonstrated increased NF-κB by enhancing IKKβ activity, which increased IκBα phosphorylation and eventual destruction [143]. Conversely, in cancer cells, including leukemia cells, Nrf2 activation does not inhibit NF-κB but acts synergistically, ultimately leading to cancer progression and resistance to treatment. In this case, a combination of antioxidant compounds and anti-inflammatory drugs can be helpful. P65 plays a dual role in modulating Nrf2 activity; specific cell types increase Nrf2 protein levels and enhance target gene expression in response to TNF-α[144, 145]. Therefore, Nrf2 and NF-κB are two essential transcription factors that control cellular oxidative stress and inflammation responses. Many diseases, including neurodegeneration, autoimmunity, and tumors, are linked to an imbalance between the Nrf2 and NF-κB pathways [146].

##### Functional interaction of Nrf2 with inflammatory mediators

In oxidative stress, activation of NF-κB leads to pro-inflammatory cytokines that stimulate the NF-κB production loop; Nrf2 negatively affects this cytokine production and NF-κB activation. In the colons of *Nrf2-null* mice, increased expression of IL-1, IL-6, iNOS, and COX2 cytokines, but Nrf2 activity suppresses their expression [147]. Using ChIP-seq and ChIP-qPCR, the researchers showed that binding Nrf2 to the IL-1 and IL-6 cytokine promoter inhibits the recruitment of RNA Pol II and cytokine transcription [129]. In addition, researchers in the experimental model of autoimmune encephalitis found that Nrf2 inhibited Th1, Th17, and Il-17 expression and improved disease process outcomes [148].

##### Role of Nrf2 in the regulation of cell adhesion molecules and regulation of protease/antiprotease balance

CAMs are cell surface receptors that bind to cells or the extracellular matrix. They play a role in cell recognition, activation, signaling, migration, proliferation, and differentiation. ICAM-1 and VCAM-1 are prominent members of the immunoglobulin superfamily among the CAMs. ICAM-1 expression and promoter activity of VCAM-1 are both inhibited by Nrf2 [149]. Several CAMs, including CD-14, TREM1, SELE, SELP, and VCAM-1, had considerably higher pulmonary expression in Nrf2^−/−^ mice [143]. In addition, cell proliferation, migration, differentiation, wound healing, angiogenesis, apoptosis, and tumor metastasis are all regulated by MMPs, which are abundant in the extracellular matrix. In inflammatory bowel disease, Nrf2/HO-1expression decreased MMP-9 and MMP-7, finally helping to reduce inflammation; The skin damage caused by UV irradiation is more severe in Nrf2 knockout mice than in WT mice, and MMP-9 and MIP-2 (macrophage inflammatory protein-2 is a crucial modulator of neutrophil recruitment) levels are much higher [150, 151]. In chondrosarcoma cells, Nrf2-knockout promotes the production of pro-inflammatory cytokines (TNF-, IL-6, and IL-1), MMP1, MMP3, and MMP13 in an osteoarthritis model [152]. As a result, the Nrf2 pathway affects protease/antiprotease balance directly or indirectly through the Nrf2-influenced NF-κB pathway in inflammation. In addition, Nrf2 reduces adhesion molecules and migration and controls the expression of proteases in normal cells, which reduces inflammation.

##### Role of Nrf2 in inflammasome signaling and Nrf2-dependent anti-inflammatory drugs

The NLR family's pyrin domain-containing 3 (NLRP3) inflammasome is a protein complex that acts as a pathogen recognition receptor (PRR) and recognizes a variety of microbial and oxidative stress signals, including pathogen-associated molecular patterns (PAMPs), damage-associated molecular pattern molecules (DAMPs), and reactive oxygen species (ROS). The stimulated NLRP3 inflammasome causes caspase-1 to cleave and the pro-inflammatory cytokine interleukin-1 (IL-1β) to be secreted, which leads to pyroptosis, a type of cell death that defends hosts from a variety of pathogens. NQO1 (an Nrf2 target gene) inhibits NLRP3 inflammasome activation, NLRP3 inflammasome assembly, caspase-1 cleavage, and IL-1β generation in macrophages. Furthermore, tert-butylhydroquinone (tBHQ) and dimethyl fumarate (DMF), both are Nrf2 activators, inhibit NLRP3 transcription [153, 154]. Even though Nrf2 inhibits NLRP3 function, it activates the NLRP3 and AIM2 inflammasomes. The Nrf2-knockout mouse macrophages showed impaired activation of the NLRP3 and AIM2, but not the NLRC4 inflammasome, according to Haitao Wen and colleagues [155]. Since Nrf2 is a key player in reducing inflammation, anti-inflammatory drugs like 2-benzyllawsone, itaconate, fumarate, and herbals contain curcumin, isothiocyanates, and anthocyanins, as well as nonsteroidal anti-inflammatory drugs like Aspirin and Celecoxib, work by activating Nrf2. These drugs reduce inflammation by activating Nrf2 [145, 150].

#### Role of Nrf2 in the regulation of genome instability

Controlling the concentration of intracellular ROS is one of the most critical issues in maintaining genomic instability. Nrf2 neutralizes ROS, while Nrf2 levels decline with age. As a result, if ROS are not effectively removed, oxidative stress will enhance and trigger the disease. Usually, aging has been linked to a decline in cell signaling, increased protein dysfunction/misfolding/aggregation, and an elevated risk of cancer and cell death. Increased expression of Nrf2's negative regulators and a general decrease in Nrf2 protein expression and pathway responsiveness have been attributed to Nrf2's steady decline with age [156, 157]. Cancer is widely considered to be an age-related disease. An increased frequency of DNA damage/replication errors in aged cells, low-dose carcinogen exposure, and variations in the levels of certain pro- and anti-tumorigenic proteins are all contributing causes (e.g., p53) [156]. Genomic instability in cancer is characterized by a lack of DNA damage repair, which results in an increased frequency of mutations and chromosomal abnormalities, similar to aging. During DNA duplication, replication errors become more common as one gets older. Because the base excision repair machinery is less effective, neurological and cancer-causing mutations are more likely. Nrf2's target genes, including p53-binding protein (53BP1) and DNA repair protein RAD51 homolog 1, play essential roles in both Homologous Recombination (HR) and Non-Homologous End Joining (NHEJ) (RAD51). By binding to the exposed ends of broken DNA strands and limiting the recruitment of repair complexes, 53BP1 promotes NHEJ by preventing the resection stage of HR. It has been shown (in vitro and in vivo) that Nrf2 activation by synthetic triterpenoids is a viable candidate target for protecting the gastrointestinal tract against acute ionizing radiation (IR) [158]. When 53BP1 promotes NHEJ by blocking the HR resection step, the Ku dimeric protein complex attaches to the damaged ends of the DNA, resulting in strand ligation.

RAD51, on the other hand, is an essential protein in HR because it forms a nucleoprotein filament on DNA that helps in the pairing stage of homologous strands. It has also been demonstrated that inhibiting Nrf2 in A549 or MCF7 cells resulted in a significant slowdown in DNA repair compared to respective radiation controls. Also, the presence of persistent DNA damage in the presence of free radical scavenger N-acetyl cysteine revealed that Nrf2's role in DNA repair was unrelated to its antioxidant capabilities [159]. These Nrf2 target genes aid DNA repair in both studies, which impedes aging and cancer. So, downregulation of Nrf2 abrogates DNA repair, which leads to a rise in overall mutations. Other studies have also highlighted the role of Nrf2 in gene instability; for example, in comparison to wild-type mice, Nrf2 knockout mice had a significantly higher incidence of gastric neoplasia caused by benzo[a]pyrene [160]. Also, researchers found that various oxidative stress and anti-oxidative stress genes are highly altered in myeloproliferative neoplasms (MPNs) using whole blood transcriptional profiling of Philadelphia-negative CML [161].

As previously mentioned, Nrf2 target genes are essential in both the HR and NHEJ parts of DNA repair, which are critical in reducing genomic instability, which is a significant cause of cancer. Nrf2 target genes are also essential mediators of xenobiotics and drug metabolism, which helps convert hazardous xenobiotics into more minor toxic forms, reducing carcinogen buildup and cancer. Nevertheless, it seems that Nrf2 plays a different role after cancer initiation and in higher stages of cancer.

### Pros and cons of the available strategies for targeting Nrf2

There is ample evidence that Nrf2 activation can reduce carcinogenesis, particularly in its early stages. Mice lacking Nrf2 are more prone to redox changes and drug toxicity [162]. However, constitutive Nrf2 activation leads to the growth of different malignancies and significantly enhances cancer resistance. Also, excessive Nrf2 expression is always associated with a poor clinical outcome [163].

Nrf2 suppression in tumor cells disrupts tumor metabolism, prevents ROS detoxification, and enhances sensitivity to chemotherapeutic drugs and radiotherapy. Moreover, Nrf2 suppression in cancer cells and healthy tissues increases sensitivity to carcinogens and impairs anti-tumor immune responses [69]. Cancer cells have abnormal Nrf2 activation for numerous advantages. Autophagy boosts cancer growth and survival by saving intracellular components to provide metabolic substrates, thus boosting nutrient starvation resistance. In turn, Nrf2 increases autophagy inhibition adaptation in cancer cells by increasing the production of proteasome subunits [164]. Also, it promotes tumor vascular endothelial development by activating and maintaining the hypoxia-inducible factor (HIF-1) pathway [112]. Nrf2 has also been linked to the epithelial-mesenchymal transition in some studies [40]. As a result, targeted Nrf2 inhibition would be the most crucial strategy for reversing the pro-tumoral impacts of constitutive Nrf2 activation.

Available strategies for targeting Nrf2 include direct Nrf2 inhibitors (inhibition of protein synthesis, inhibition of Nrf2 transcriptional activity, inhibition of Nrf2 accumulation in the nucleus by Trigonelline, inhibition of P62-Keap1 association, inhibition of PI3K/AKT, inhibition of FN3K, and activation of the Brain-Specific Kinase 2), and alternative ways to target Nrf2/Keap1 mutant cancers (direct inhibition of the cystine/glutamate antiporter system, inhibition of the Pentose phosphate pathway, NQO1 bioactivatable drugs, and Aldo–Keto Reductases) [69].

In summary, researchers previously concentrated on designing different therapeutics for cancer patients with Nrf2 stimulation by finding specific Nrf2 inhibitors. Recent studies showed that systemic suppression of Nrf2 could promote tumor growth. More targeted and effective inhibitors and more mechanistic research are required to understand how Nrf2 suppression affects both Nrf2-addicted cancer cells and healthy cells in the microenvironment.

### Nrf2 in leukemia

Shibata et al*.* identified somatic mutations in Nrf2 in different tumors such as lung, head and neck, adenocarcinomas, and large cell neuroendocrine carcinomas [165]. Mutations in the Nrf2 genes can promote solid tumors and hematological malignancies. Moreover, Nrf2 mutations may reduce the sensitivity to chemotherapy and radiotherapy. However, more studies are needed to clarify the role of Nrf2 in leukemia progression.

Our main aim in this study is to determine the role of this transcription factor in different types of leukemia (Table 1) and whether Nrf2 would be a suitable target to treat these malignancies.

**Table 1:** Nrf2 in leukemia

Type of leukemia	Type of study	Cell line	Study results	Refs
CML	In vitro	K-562 and KU-812	Nrf2 gene targets such as HO-1 and NQO1 increased imatinib resistance and decreased apoptosis in CML	[176]
CML	In vitro	K562/G01	When Nrf2 expression was inhibited by siRNA, reactive oxygen species (ROS) and the rate of apoptosis in response to imatinib increased, and cell proliferation decreased	[177]
CML	In vitro	HL60/A(AML) and K562 /G(CML)	Using Triptolide, a natural inhibitor of Nrf2, along with other drugs such as doxorubicin and imatinib, reduced HIF-1a, Nrf2, and drug resistance	[178]
CML	In vitro and in vivo	K562/A02 and NOD/SCID mice	Wogonin, an Nrf2 inhibitor, reduced Adriamycin resistance by inhibiting the Stat3/NF-κB—signaling	[179, 180]
CML	In vitro	K562	Chaetominine disrupted the PI3K/Akt/Nrf2 signaling pathway, inhibited the MRP1-mediated drug efflux pump, induced Bax apoptotic protein, and inhibited anti-apoptotic proteins	[181]
AML	In vitro	Blood samples from 15 AML patients (PBMC)	Malignant AML stem cells increased NF-κB expression, responsible for high Nrf2 expression	[144, 186]
AML	In vitro	THP-1, HL60, U937, and AML 193	In cells resistant to TNF-induced cell death, the Nrf2 pathway is activated	[187]
AML	In vitro	17 patients with AML and THP-1, HL60, and U937	1) Simultaneous inhibition of HO-1 and NF-κB might be fluent in increasing apoptosis 2) high expression of Nrf2 as a result of NF-κB expression	[91]
AML	In vitro and in vivo	THP-1, HL-60, U937, and CAMs of chicken eggs	4f drug-based therapy induces programmed cell death (dependent on mitochondria) by decreasing the level of Nrf2 protein and increasing caspase-3, cleaved poly (ADP-ribose) protein levels, a pro-apoptotic protein. Also, tumor growth was inhibited by 4f in a chick embryo model	[188]
AML	In vitro	THP-1 and U937	Several Nrf2 inhibitors were identified, including ATRA, brusatol, and luteolin, which sensitized cells to arsenic trioxide (As2O3), etoposide, and doxorubicin	[189]
AML	In vitro	HL60, Molm13, THP-1, and U937	Nrf2 inhibitor, Cytarabine, and Daunorubicin decreased drug resistance in AML	[190]
AML	In vitro	KG1	Leukemia stem cells are resistant to apoptosis by activating the PERK/Nrf2 signaling pathway	[191]
AML	In vitro and in vivo	KG1α, Kasumi-1 and NOD/SCID mice	Disulfiram/copper, which had an inhibitory effect on NF-κB and Nrf2, killed the malignant stem cells in AML	[192]
AML	In vitro	ALL (REH, MOLT4) AML (MOLM-14)	Inhibition of MAPK/ERK and PI3K/AKT pathways reduced the expression of Nrf2, which was associated with downregulation of target genes, upregulation of ROS, and increased apoptosis	[193]
AML	In vitro	U937 HL60	Nrf2 activators in AML cell line U937 prevented the toxicity of calcium (dimethyl fumarate (DMF), tert-butyl hydroquinone, or carnosic acid	[194, 195]
AML	In vitro	U937, MOLM-13, HL-60, THP1, KG1a	Combination therapy with cytarabine (AraC), DHA, and EPA in AML cell lines increased cell cytotoxicity	[196]
APL	In vitro	PR9 (U937 cell line with zinc inducible PML/RARa expression)	1) Nrf2 expression in APL is lower than in AML because some inhibitory mechanisms hinder Nrf2 transcription activity 2) suppressing Nrf2 activity in APL made cells sensitive to treatment with high doses of ascorbate	[199]
APL	In vitro	NB4	NF-κB increased Nrf2 expression and leukemia progression	[200]
CLL	In vitro	Blood samples (PBMC)	The PBMC of patients with CLL had higher levels of Nrf2 than normal blood samples	[203]
CLL	In vitro	Blood samples (PBMC)	Increased ROR1 expression in CLL cells increases APRIL and BAFF-R expression, leading to the recruitment and accumulation of the p62 protein, which triggers several separate signaling pathways such as Nrf2	[204]
ALL	In silico	–	The Nrf2 inhibitory pathway and activation of this factor are disrupted in patients with ALL	[205]
ALL	In vitro	REH, MOLT-4	Inhibition of MAPK/ERK and PI3K/AKT pathways reduced Nrf2/NF-κΒ and drug resistance The combination of MAPK/ERK pathway inhibitors plus topoisomerase II inhibitor treatment synergistically increased the production of ROS and caused apoptosis in leukemic cells	[193, 207, 208]

In 2012, about 350,000 new cases of leukemia (2.5 percent of all new cancer cases) were reported. The lowest leukemia incidence rate was observed in Middle and West Africa (less than 3 per 100,000 males and less than 2 per 100,000 females). Besides, the highest incidence rate was reported in North America and Australia/New Zealand (more than 10 per 100,000 males and 7 per 100,000 females) [166, 167]. Leukemia is more common in Western countries, and men are more at risk than women [168]. It is supposed that different environmental, genetic, and epigenetic factors play essential roles in leukemia occurrence [169–171].

Nrf2 increases the expression of drug efflux pumps due to its reducing ROS and neutralizing electrophilic compounds. This action excretes drug chemicals from malignant cells and inhibits the accumulation of drugs within cells. Nrf2 also enhances the expression of anti-apoptotic proteins BCL2 and NF-κB Interplay in regulating the cellular redox pathway, which promotes cell survival. Nrf2 can regulate enzymes involved in drug metabolism, including GST, UDP-glucuronosyltransferases, and NQO1, and causes chemotherapy resistance [88, 144, 172, 173]. In the following, we will discuss the role of Nrf2 in leukemia and try to determine the role of this transcription factor in survival, apoptosis, metastasis, and other cellular metabolic pathways.

#### Nrf2 and Chronic myeloid leukemia (CML)

CML begins in specific bone marrow hematopoietic cells (myeloid cells) that generate red blood cells, platelets, and most white blood cells (except lymphocytes). In CML, a genetic change results in a chromosomal translocation between chromosomes (9–22), also known as the Philadelphia chromosome. BCR/Abl, oncogene originated from this fusion which encodes a 210 kDa oncoprotein. Over time, malignant cells can spread to other body parts, including the spleen. CML is relatively slow but can progress rapidly to acute leukemia, making it challenging to treat. CML is more common in adults but also rare in children [174, 175].

Bonovolias et al*.* demonstrated that Nrf2 targets like HO-1 and NQO1 suppressed the anti-apoptotic Bcl-2a and Bcl-2b genes, which increased drug resistance to imatinib and reduced apoptosis in K-562 and KU-812 human CML cell lines [176]. Therefore, Nrf2 targeting increased the sensitivity of the leukemic cells to imatinib. Additionally, in a study by Xu et al*.,* the expression of Nrf2 and its target gene TrxR reduced the sensitivity of the K562/G01 imatinib-resistant cell line to imatinib therapy.

Interestingly, Nrf2 silencing in the K562/G01 cell line with specific siRNA enhanced ROS generation, reduced cell proliferation, and increased imatinib-induced apoptosis [177]. It has also been shown that the extract of the Chinese plant Triptolide (TPL), which has been used to treat autoimmune diseases such as rheumatoid arthritis, asthma, and transplant rejection, could be a natural inhibitor of Nrf2. Also, It has been shown that the combination of TPL with DOX and imatinib could reduce HIF-1α, Nrf2 expression, and drug resistance in HL60/A (AML) and K562/G (CML) cell lines. The mRNA level of both HIF-1α and Nrf2 was measured by Western blot and RT-PCR methods to confirm the effect of TPL in reducing their expression. The results showed that resistant cell lines had higher levels of HIF-1α and Nrf2 compared to treated cell lines. Interestingly, TPL-treated cell lines not only had lower HIF-1α levels, but HIF-1α downstream genes, including BNIP3, VEGF, and CAIX, were also downregulated [178]. Moreover, Wogonin (5,7-dihydroxy-8-methoxy flavone), a natural compound of the flavonoids group, categorized as an Nrf2 inhibitor, is extracted from the root of Scutellaria baicalinase’s Georgi and is considered an anti-cancer agent. Wogonin has multiple functions like phosphorylation of IκBα and IKKβ, reducing p65 and Nrf2 levels, suppressing NF-κΒ activity and blocking STAT3 and P3IK signaling pathways. Wogonin can also reverse multidrug-resistant response (MDR), the major problem in CML therapy failure. It has been shown that treatment of the K562/A02 CML cell line by the Nrf2 inhibitor, Wogonin, is associated with a blockade of the Stat3/NF-κB signaling pathway and a reduction of Adriamycin resistance. The studies on the Adriamycin-Resistant (ADR) K562/A02 cell line found that NF-κΒ expression levels were high, which led to the further expression of anti-apoptotic proteins, secretion of pro-inflammatory cytokines, and increasing MDR phenotype. High expression of Nrf2 concerning HO-1 causes expression of NF-κΒ and AP-1. To better understand the relationship between Nrf2 and NF-κΒ transcription factors, K562/A02 cell line was treated with an NF-κΒ activator (LPS), which was associated with an increase in the Nrf2 expression level [179, 180].

Furthermore, NOD/SCID immunodeficient mice were inoculated with K562 and K562/A02 cells. The transplanted animals then received ADR (4 mg/kg) intravenous injections twice a week for 4 weeks, followed by Wogonin (40 mg/kg) intravenous injections every other day. Flow cytometry was used to detect CD13^+^ cells in the BM, PB, and spleen. The combination group showed a significant reduction in CD13^+^ cells in BM and PB cells. Organs (spleen and liver) were also collected and weighed from the animals. The spleen of the control group showed normal histology, with splenic parenchyma composed of normal white and red pulp and a typical splenic capsule and trabeculae, whereas the liver of the leukemia-induced group demonstrated invasion by neoplastic cells and multiple foci of extramedullary hematopoiesis. The liver histology was normal after treatment with ADR and Wogonin [179].In a further study on Nrf2 to find novel therapeutic strategies in leukemia, Yao et al*.* introduced chaetominine as a cytotoxic alkaloid that could disrupt the PI3K/Akt/Nrf2 signaling pathway. Chaetominine also inhibited the MRP1-mediated drug efflux pump, blocked the anti-apoptotic protein Bcl2, induced Bax apoptotic protein, and reduced drug resistance to Adriamycin (ADR). Notably, chaetominine dramatically reversed drug resistance and decreased drug efflux in the K562/Adr leukemia cell line by repressing the PI3K/Akt/Nrf2 pathway. Dysregulation of the PI3K/Akt/Nrf2 axis decreased MRP1, which increased intracellular ADR accumulation [181].

Most studies have shown an increase in Nrf2 expression in CML cases. Recently, suppression of this transcription factor has been proposed as a new therapeutic target to overcome chemotherapy resistance and increase the apoptosis rate in malignant cells. For this reason, suppressing Nrf2 expression through various inhibitory pathways such as siRNA technology and natural compounds (TPL, Wogonin, chaetominine) could help decrease drug resistance in CML.

#### Nrf2 and Acute myeloid leukemia (AML)

AML accounts for one-third of all cases of acute leukemia in childhood and adolescence and is more common in middle age (60 years) [182]. This malignancy is caused by genetic disorders and other risk factors that affect the myeloid progenitor stem cells [183–185]. Leukemic stem *cells* (LSCs) (CD34^+/^CD38^−/^CD123^+^) have the power of self-renewing and limitless proliferation, and as they grow, they produce large numbers of leukemic cells. LSCs in AML have a high expression of NF-κB, but normal unstimulated stem cells do not express this factor. Therefore, targeting NF-κB is a suitable therapeutic target in leukemia. A study on the blood samples derived from 15 AML patients and the electrophoretic mobility shift assay (EMSA) detected that malignant AML stem cells have NF-κB binding activity not seen in normal stem cells. It is demonstrated that this NF-κB binding activity confirmed Nrf2 overexpression in LSCs. In that study, researchers used proteasome inhibitors (MG-132) that inhibited the degradation of NF-κB suppressors (IκBα). This proteasome inhibitor decreased NF-κB activity and increased IκBα. In malignant cells, NF-κB reduction induced apoptosis and reduced drug resistance but did not affect normal cells [186]. In a study on AML cell lines, including THP-1, HL60, U937, and AML193, these cells were resistant to TNF-induced cell death. Activation of the Nrf2 pathway induced the production of HO-1 and NF-κB, making cells resistant to apoptosis and TNF-induced cell death. Cell lines were exposed to the TNF cytokine to induce apoptosis, while the NF-κB pathway was inhibited by BAY 11–7082. Interestingly, TNF induced death cell signaling or amplified growth signal by binding to TNFR1 and TNFR2 [187]. Besides, a study on 17 patients with AML and a human AML cell line, and THP-1, demonstrated that despite the high NF-κB expression in AML patients, inhibition of this factor (with BAY 11–7082) did not significantly increase the rate of apoptosis. The researchers proved that the simultaneous inhibition of HO-1 and NF-κB might effectively increase apoptosis. On the other hand, the level of HO-1 mRNA was increased in cell lines treated by BAY 11–7082. This study confirmed the presence of another regulatory pathway (supposed to be Nrf2) that controls the process of chemotherapy resistance and apoptosis [91]. Moreover, Rushworth et al*.* introduced the high expression of Nrf2 due to the NF-κB expression. This study proved NF-κB as a factor in chemotherapy resistance, enhancing leukemia's progression in overexpression cases. Furthermore, Rushworth et al. determined the regulatory effects of NF-κB on Nrf2. NF-κB and Nrf2 mRNAs were reduced in BAY 11–7082 treated THP-1, HL60, and U937 cell lines. Also, chemotherapy administration in Nrf2 knockdown cells decreased colony formation ability and enhanced apoptosis [91]. Many studies also had similar results in HL60, THP-1, and U937 cell lines [144, 187]. Zhang et al*.* evaluated the effect of the Nrf2 inhibitor, pyrazolyl hydroxamic acid derivative (4f), on three AML cell lines, including THP-1, HL-60, U937, and chick embryo model. In this study, 4f could reduce cell line growth in a dose-dependent manner so that with increasing drug concentration, cytotoxicity was increased. The rate of apoptosis and necrosis of cell lines treated with 4f was assessed by flow cytometry. The results demonstrated an increase in apoptosis levels in treated cell lines. Also, the effect of the Nrf2 activator, tert-butylhydroquinone (TBHQ), on cell lines was investigated; TBHQ therapy increased Nrf2 protein levels and cell viability while decreasing C-caspase-3 and C-PARP (poly-ADP-ribose-polymerase). Therefore, 4f drug-based therapy-induced programmed cell death (dependent on mitochondria) decreases the level of Nrf2 protein and increases caspase-3 and cleaved poly (ADP-Ribose) protein levels. On the other hand, it increased the expression of Bax, a pro-apoptotic protein. In addition, the researchers seeded AML cells on chicken egg CAMs and assessed the tumor-growth-inhibitory effect of compound 4f therapy in vivo. Data demonstrated that 4f treatment reduced tumor size in CAMs compared to controls, and 4f-treated tumor sections had an increased number of apoptotic cells compared to controls [188]. Peng et al*.* showed that knockdown of Nrf2 by lentiviral shRNA or Nrf2 inhibitors such as isonicotinic acid amides, isoniazid, and ethionamide in THP-1 and U937 cell lines, could enhance the effect of chemical compounds, including arsenic trioxide (As2O3), etoposide, and doxorubicin. On the other hand, in this study, several Nrf2 inhibitor molecules were identified, including ATRA, brusatol, and luteolin [189]. In another study, Karathedath et al*.* used Bristol, an Nrf2 inhibitor, in the AML human cell lines (HL60, Molm13, THP-1, and U937) along with Cytarabine (Ara-C) and Daunorubicin (Dnr), to decrease drug resistance and enhance apoptosis in AML cells. They showed that brusatol reduces colony formation and Nrf2 protein levels [190]. It is also demonstrated that LSCs activate the PERK/Nrf2 signaling pathway and, through this pathway, inhibit apoptosis induction. PERK/Nrf2 pathway silencing by specific siRNAs in the KG1 leukemic cell line increased susceptibility to apoptosis. It is confirmed that G9a (a new histone methyltransferase; HMTase) overexpression is associated with poor survival and enhances disease progression, metastasis, and chemotherapy resistance. This study highlights the role of epigenetic modifications in leukemia and suggests PERK/Nrf2 pathway inhibition as a potential therapeutic approach in AML. It has been shown that G9a inhibition with BIX-01294 in U937 and KG1 cells suppresses the PERK/Nrf2 signaling pathway and induces apoptosis in these cell lines. Thus PERK/Nrf2 signaling attenuated the BIX-01294 mediated effects like ROS generation and apoptosis. Combination therapy of U937 and KG1 cell lines by BIX-01294 and PERK/Nrf2 pathway inhibitors also increased C-caspase3 and C-PARP levels and induced phosphorylation of p38. Furthermore, flow cytometry results showed increased levels of apoptosis in cell lines that were treated [191]. In another study, Xu et al*.* used Disulfiram/copper (DS/Cu), which inhibited NF-κB and Nrf2 factors, simultaneously activated the ROS-JNK pathway, and destroyed malignant stem cells in AML. The results of the MTT assay for DS on KG1α cells showed an increase in cell proliferation with an IC50 value of 0.54 ± 0.18 μM. Moreover, the combination therapy of DS and Cu (DS/Cu) reduced the IC50 value to 0.21 ± 0.03 μM. Cell lines exposed to (DS / Cu) were also examined at different times by a DCFH-DA-based assay. Results showed an increase in ROS levels in treated cells. Thus, it was proved that DS/Cu specifically targets LSC in AML, inhibits proliferation, induces apoptosis, and suppresses colony formation in LSCs [192]. Moreover, researchers analyzed the anti-cancer effects of DS/Cu in vivo by injecting CD34^+^/CD38 KG1 cells into SCID mice to produce a leukemia mouse model. Tumor development was significantly slowed after DS/Cu therapy [192]. Further investigation showed that the MAPK/ERK and PI3K/AKT pathways stimulate NF-κB and Nrf2. The researchers discovered that inhibiting the NF-κB and Nrf2 pathways with the ERK1/2 inhibitor AZD0364 and the PI3K inhibitor ZSTK474 could reduce the expression of Nrf2 and ROS in ALL (REH, MOLT-4) and AML (MOLM-14) cell lines [193].

Conversely, vitamin D derivatives differentiate immature cells in AML, but high doses of these derivatives cause calcium toxicity and deposition in the kidneys. Researchers have used Nrf2 activators, including dimethyl fumarate, tert-butyl hydroquinone, or carnosic acid, which are used as adjuvants and vitamin D to reduce its dose in the U937 AML cell line to prevent the toxicity of calcium [194, 195]. Pico et al*.* also showed that the increased expression of Nrf2 in the AML cell line exposed to docosahexaenoic acid (DHA) and eicosapentaenoic acid (EPA) was associated with changes in mitochondrial metabolic pathways and malignant cell apoptosis. In treated cell lines with DHA and EPA, mitochondrial energy metabolism changed from oxidative respiration to glycolytic metabolism during the Warburg effect, which led to upregulation of Nrf2 and ROS levels, downregulation of COX and PGE2 (decrease metastasis), inhibition of NF-κB and PI3K/Akt pathway, and suppression of angiogenesis. Besides, combination therapy with cytarabine (AraC), DHA, and EPA in the AML cell line increased cell cytotoxicity [196].

In summary, most studies have shown that increased expression of this transcription factor exacerbates the disease and causes chemotherapy resistance. So, targeting this biomarker with various inhibitors reduces resistance and induces apoptosis.

However, a few conflicting studies suggest that increasing Nrf2 expression is influential in treating AML. Therefore, more investigation is needed to clarify the role of Nrf2 in AML.

#### Nrf2 and Acute promyelocytic leukemia (APML, APL)

Acute promyelocytic leukemia (APML, APL) is a subtype of acute myeloid leukemia (AML) associated with an abnormal accumulation of immature granulocytes called promyelocytes in the bone marrow and blood. 95% of APL cases are characterized by a reciprocal chromosomal translocation of t(15;17) (q24; q21) [197, 198]. Banella et al*.* studied the PR9 cell line (a U937-derived cell line containing zinc-inducible PML/RARA) and Mock control cells (a PML/RARA-lacking U937 cell line). They revealed that Nrf2 expression in APL is lower than in AML because some inhibitory mechanisms hinder Nrf2 transcription activity. On the other hand, suppressing Nrf2 activity in APL made cells sensitive to treatment with high doses of ascorbate. In this study, researchers found that the Nrf2 level declines and the PML-RARα fusion protein increase in APL [199]. It has been shown that high doses of ascorbate preferentially induce apoptosis in leukemic blast cells derived from APL patients. This study also defined various mechanisms involved in leukemic blast cell killing, like inhibition of Nrf2 function by PML/RARa, and impediment of Nrf2 translocation to the nucleus through enhancing the PML/RARa-Nrf2 attachment and elevating Nrf2 degradation in the cytoplasm in a Keap1- dependent manner [199].

Rubio et al. demonstrated the role of NF-κB in increasing Nrf2 expression on the NB4 leukemic cell line, which was associated with disease progression. This study introduced the role of two antioxidant compounds (Quercetin and Esculetin) on the leukemic cell line. They found that Quercetin had a suppressing effect on the proliferation and growth of the leukemic cell lines. Quercetin activated caspase protein and mitochondrial-dependent cell death apoptosis pathways in leukemic P39 cells through downregulation of anti-apoptotic proteins (Bcl2, Mcl-1, and Bcl-xl) and stimulation of pro-apoptotic protein (Bax). Escalation is a tyrosinase inhibitor that induces apoptosis in U937, HL-60, and NB4 (APL cell line) cells. They investigated the effects of Quercetin and Esculetinon inhibitors on the NB4 cell line alone and in combination. The study showed that treated cell lines with Quercetin had elevated levels of Nrf2 and decreased levels of NF-κB in the cytosol. Also, the level of Nrf2 in the nucleus decreased. In treated cell lines with Esculetin, the Nrf2 level in the nucleus increased. Interestingly, the combined effect of Quercetin and Esculetin showed elevation of Nrf2 and NF-κB in the nucleus. On the other hand, the NF-κB inhibitor reduced Nrf2 expression in treated cells [200].

In summary, various methods for reducing Nrf2 expression in APL cells exist. Compared to other AML subtypes, APL is more sensitive to chemotherapy. Studies propose the connection between Nrf2 and NF-κΒ, and targeting these factors can be considered effective for increasing the survival rate of patients with APL and their response to chemotherapy and other leukemia.

#### Nrf2 and chronic lymphocytic leukemia (CLL)

Chronic lymphocytic leukemia (CLL) is the most common adult leukemia in the Western world. It is a type of cancer that chronically affects the progenitor lymphoid lineage of bone marrow cells [201, 202]. Many people have no symptoms for at least a few years and manage their disease by regular monitoring without treatment. Nevertheless, the cells grow and spread over time to other body parts, like the lymph nodes, liver, and spleen [88].

It has been demonstrated that compounds such as unsaturated carbonyls, sulfhydryl reactive metals, and isothiocyanates significantly induce Nrf2 in CLL peripheral blood mononuclear cells (PBMCs).

Furthermore, qPCR and immunoblot analysis of CLL patients' PBMCs revealed that they had higher levels of Nrf2 than normal blood samples. Furthermore, the results showed that the α-β unsaturated carbonyl functional group is necessary for Nrf2 activation and cell cytotoxicity [203]. A recent study by Lopez et al*.* showed that BAFF production by nurse-like cells (NLC) increases Receptor Tyrosine Kinase Like Orphan Receptor 1 (ROR1) receptor expression in CLL cells. The increased ROR1 enhanced A proliferation-inducing ligand (APRIL) and B cell activation factor receptor (BAFF-R) or BLyS receptor 3 (BR3) expression. Downstream signaling of these pathways leads to the recruitment and accumulation of the p62 protein (autophagy and ubiquitin signaling adapter, upregulating mTORC1 signaling), which triggers several signaling pathways for Nrf2 expression (Fig. 2). Nrf2 overexpression reduced the ROS level, induced chemo-resistance, and activated the mTORC1 metabolic pathway and NF-κB pathway (by inducing p65 phosphorylation). Identically, BAFF incitement in CLL cells decreased mt-ROS accumulation. This process indicated the importance of the tumor microenvironment. They demonstrated that IKKβ inhibitors, the responsible kinase for canonical NF-κB signaling, increased inflammation due to NLRP3 overactivation and led to disease progression. Also, p62 erosion stimulated NLRP3 inflammasome activation. Humanized Cirmtuzumab antibody, which inhibits ROR1, was used to block these signaling pathways and had fewer side effects [204].

In summary, tumor microenvironments promote the progression of chronic lymphocytic leukemia (CLL) and chemotherapy resistance, making it challenging to design the correct treatment. Moreover, Nrf2 is elevated in the CLL microenvironment and plays an essential role in leukemia cell survival. However, few studies are available on the role of Nrf2 in CLL, so future studies are needed to elucidate the role of this transcription factor completely.

#### Nrf2 and acute lymphocytic leukemia (ALL)

Acute lymphocytic/ lymphoblastic leukemia (ALL) can progress rapidly and, if left untreated, can be fatal within a few months. ALL is commonly seen in children. This type of leukemia involves the early (immature) forms of lymphocytes and has different subtypes that cause malignancy in B and T cells. The Philadelphia (Ph ') chromosome, which is formed during the translocation of chromosomes (9; 22) (q34; ql1), most often manifests itself in CML and some patients with ALL (5 to 20%) [72, 205]. ALL are routinely treated with cytosine arabinoside, doxorubicin, cyclophosphamide, and methotrexate. It is demonstrated that upregulation of Nrf2 enhances chemotherapy resistance and causes the survival of malignant cells. Besides, gain-of-function mutations of Nrf2 and loss-of-function mutations of Keap1 cause several cancers and provoke drug and radiation resistance. In an in-silico study by Akın-Balı et al., 6 to 8 new mutations in the Nrf2-Keap1 signaling pathway were identified, indicating a disruption of the Nrf2 inhibitory pathway and activation of this factor in patients with ALL. In this study, 30 ALL patients (20 girls and 10 boys) aged between 1 and 14 years (mean age ¼ 5.4 years) participated. Through DNA sequencing by PolyPhen-2 program analysis, pathological mutations were recognized in NF-κB1, Keap1, and p62 pathways. Analysis of mutations showed 18 changes (11 nucleotide substitutions, three deletions, and four insertions). These changes were detected in 22 (73%) of 30 pediatric ALL patients [206].

The mitogen-activated protein kinase (MAPK)/extracellular signal kinase (ERK) and phosphatidylinositol 3-kinase (PI3K)/protein kinase B (AKT) signaling pathways are essential pathways that take part in numerous cellular functions, like cell durability, proliferation, differentiation, drug resistance, and apoptosis. Overactivation of these pathways is prevalent in many cancers, such as leukemia, which are significant causes of cancer drug resistance. Thus, targeting this signaling pathway can suppress cell proliferation and induce apoptosis. Gajda et al. demonstrated that inhibiting the MAPK/ERK and PI3K/AKT pathways reduces Nrf2/NF-κΒ levels and prevents drug resistance in REH and MOLT-4 ALL cell lines [193]. Furthermore, a combination of MAPK/ERK pathway inhibitors plus topoisomerase II inhibitor treatment synergistically increased the production of ROS and caused apoptosis in leukemic cells [207, 208].

Increased GSH, Trx, and TrxR expression levels were seen in ALL, AML, and CLL patients, partly due to Nrf2 activation. Also, Trx initiated the PI3K/AKT pathway. In this study, researchers exposed REH, MOLT-4 (ALL), MOLM-14 (AML), and K562 (CML) cell lines to AZD0364 and/or ZSTK474. The results showed that therapy decreased cell viability in REH, MOLT-4, and MOLM-14b cells but was ineffective in K562 cells. Also, the ROS level was increased. On the other hand, expression analysis showed a decrease in NF-κB, Nrf2, HO-1, and TrxR proteins in REH, MOLT-4, and MOLM-14b cells, whereas the level of HO-1 in the K562 cell line increased. In addition, the increased apoptosis levels were confirmed by flow cytometric analysis in AML and ALL cell lines [193].

In summary, the studies showed an increased expression of Nrf2 and its target genes in ALL. On the other hand, DNA sequencing confirmed the presence of mutations in NF-κB1, Nrf2, Keap1, and p62 pathways, which disrupt the Nrf2 inhibitory pathway and cause stimulation of this transcription factor in ALL and result in decreased apoptosis in the malignant cell, and chemotherapy resistance. In addition, phosphorylation and activation of transcription factors such as MAPK/ERK and PI3K/AKT, which are located upstream or downstream of Nrf2, stimulate the expression of Nrf2/NF-κΒ. Therefore, targeting these biomarkers can be promising in treating leukemia and increasing patient survival.

## Conclusion

This review is more comprehensive than the recent papers in the field [12, 13], and it also summarizes the available evidence to evaluate the role of Nrf2 in various leukemia [209]. Nrf2 is a poor prognostic marker in leukemia [210] and affects the expression of several critical target genes in leukemia progression [92, 211, 212]. Overexpression of Nrf2 induces different signaling pathways, stimulates the accumulation of P62 protein that leads to autophagy pathways, induces cytokines, and increases various kinases' expression. Also, Nrf2 increases or decreases structural proteins, including E-cadherin, and modulates various signaling pathways such as Nrf2/NF-κΒ, MAPK/ERK, and PI3K/AKT signaling pathways inducing the expression of anti-apoptotic proteins and triggers chemotherapy resistance [41, 203].

Several studies suggest that inhibiting Nrf2-related antioxidant processes may be a practical and promising treatment strategy for inducing prooxidizing effects in the cancer cell microenvironment and triggering ROS-dependent cell death in various human cancers [213, 214]. Also, Nrf2 inhibitors and activators are used in cancer therapy and inflammatory diseases according to the type and stage of cancer [41]. Nrf2 activators play a cytoprotective role, protecting healthy cells from carcinogens. On the other hand, Nrf2 inhibitors suppress cancer cell proliferation and inhibit metastasis and angiogenesis in various myeloid and lymphoid leukemia [88, 215]. Furthermore, inhibition of Nrf2, whether by knockdown techniques, siRNA, or the use of inhibitory compounds, combined with current frontline therapies, stops cell growth and causes apoptosis in malignant cells, which should be investigated in subsequent studies [92, 216–218]. Given Nrf2's available location at the pivotal point of various pathways, pharmacologic manipulations of downstream effectors or upstream regulators of Nrf2 signaling could produce significant anti-tumor effects and synergize with already established drugs [219].

In summary, due to the function and overexpression of Nrf2 in the various types of leukemia and cancer, inhibition of this transcription factor alone or in combination with other cancer therapies such as immunotherapy or chemotherapy can be considered a novel promising therapeutic target; for example, combining therapy of CML patients with Nrf2-siRNA and imatinib enhanced the rate of apoptosis and reduced imatinib resistance [177]. Also, AML patients were less resistant to treatment when the Nrf2 inhibitor was used with cytotoxic drugs like Cytarabine and Daunorubicin [190].

So, it is likely that throughout the near future, more studies will search for other valuable ways of preventing the pro-oncogenic roles of Nrf2 and protecting its biological effects. The challenge is finding new and effective ways that don't have other targets. On the other hand, it is important to note that finding new Nrf2/ARE inhibitors with the right potency, specificity, and safety is still a significant challenge in cancer research and could lead to a breakthrough in the hard fight against leukemia and cancer.

## Data Availability

Not applicable.
